# Effects of Chinese Medicine Tong xinluo on Diabetic Nephropathy via Inhibiting TGF-**β**1-Induced Epithelial-to-Mesenchymal Transition

**DOI:** 10.1155/2014/123497

**Published:** 2014-04-17

**Authors:** Na Zhang, Yanbin Gao, Dawei Zou, Jinyang Wang, Jiaoyang Li, Shengnan Zhou, Zhiyao Zhu, Xuan Zhao, Liping Xu, Haiyan Zhang

**Affiliations:** ^1^School of Traditional Chinese Medicine, Capital Medical University, No. 10, Youanmenwai, Xitoutiao, Fengtai District, Beijing 100069, China; ^2^Department of Cell Biology, School of Basic Medical Science, Capital Medical University, No. 10, Youanmenwai, Xitoutiao, Fengtai District, Beijing 100069, China

## Abstract

Diabetic nephropathy (DN) is a major cause of chronic kidney failure and characterized by interstitial and glomeruli fibrosis. Epithelial-to-mesenchymal transition (EMT) plays an important role in the pathogenesis of DN. Tong xinluo (TXL), a Chinese herbal compound, has been used in China with established therapeutic efficacy in patients with DN. To investigate the molecular mechanism of TXL improving DN, KK-Ay mice were selected as models for the evaluation of pathogenesis and treatment in DN. In vitro, TGF-**β**1 was used to induce EMT. Western blot (WB), immunofluorescence staining, and real-time polymerase chain reaction (RT-PCR) were applied to detect the changes of EMT markers in vivo and in vitro, respectively. Results showed the expressions of TGF-**β**1 and its downstream proteins smad3/p-smad3 were greatly reduced in TXL group; meantime, TXL restored the expression of smad7. As a result, the expressions of collagen IV (Col IV) and fibronectin (FN) were significantly decreased in TXL group. In vivo, 24 h-UAER (24-hour urine albumin excretion ratio) and BUN (blood urea nitrogen) were decreased and Ccr (creatinine clearance ratio) was increased in TXL group compared with DN group. In summary, the present study demonstrates that TXL successfully inhibits TGF-**β**1-induced epithelial-to-mesenchymal transition in DN, which may account for the therapeutic efficacy in TXL-mediated renoprotection.

## 1. Introduction

Diabetic nephropathy (DN) and other chronic kidney diseases are characterized by glomeruli and interstitial fibrosis. Traditionally, resident fibroblasts are considered to be the key mediators of renal fibrosis; now, convincing evidences suggest that the appearance of interstitial myofibroblasts also contributes to fibrosis. Central to this process is epithelial-to-mesenchymal transition (EMT) [1]. Researches show that about 30% fibroblasts are derived from the tubular epithelial cells via EMT in kidney [2].

EMT is regulated by different signaling molecules and transforming growth factor *β*1 (TGF-*β*1) is proved to be the principle mediator in EMT [3]. In diabetes, high glucose and other stimuli increased the production of TGF-*β*1 [4]. TGF-*β*1 has the ability to increase its own expression leading to the accumulation of ECM and fibrosis [5]. Combined with T*β*RII, TGF-*β*1 initiates the expressions of several downstream signal proteins such as small mothers against decapentaplegic (smad) and mitogen-activated protein kinases (MAPKs). Loss of E-cadherin (E-CA) and gain in *α*-smooth muscle actin (*α*-SMA) expression are hallmarks of EMT [6]. As a result, extracellular matrix (ECM) such as collagen IV (Col IV) and fibronectin (FN) excessively accumulated. With the development of renal fibrosis, many patients may undergo end-stage renal disease (ESRD) and DN is the leading cause of ESRD, accounting for millions of deaths worldwide [7]. In treating DN and other chronic renal diseases, most therapies aim at the heteropathy such as the control of blood pressure and blood glucose.

With the development of modern technologies, preparation of Chinese medicine has also been improved. Tong xinluo (TXL), a Chinese herbal compound developed two decades ago, includes a group of medicines such as* Panax ginseng *C. A. Mey. extract and* Paeonia lactiflora *Pall. extract [8, 9]. It was registered in the State Food and Drug Administration of China. TXL has already been proven to have a cohort of potentially therapeutic value such as preventing apoptosis, improving endothelial cell function, reducing inflammation, and lowering lipids [10]. It has already been used in treating angina pectoris diseases in China for many years [8]. According to these positive effects, many studies have investigated the mechanism of it, that is, TXL modulates vascular endothelial function by inducing eNOS expression via the PI-3K/Akt/HIF-dependent signaling pathway [11]. TXL dose-dependently enhanced stability of vulnerable plaques compared with a high-dose simvastatin [12]. TXL showed protective effects on free fatty acid induced endothelial injury by boosting intracellular antioxidant capacity through AMPK pathway [8]. In treating DN, TXL also shows positive effect. Meta-analysis showed that TXL significantly decreased 24-hour urine albumin excretion ratio (24 h-UAER) and blood urea nitrogen (BUN) [13]. In the treatment of early DN, TXL could improve renal microcirculation, reduce Cys-C and UAER, and delay the progress of renal damage [14]. But the mechanism of TXL improving DN remains unclear. Fibrosis is a key pathology in DN and excessive extracellular matrix (ECM) synthesis and accumulation, resulting in glomerular and tubular pathology and ultimately death in diabetic patients [15]. Studies have proven the positive effect of TXL and some components of it in suppressing renal fibrosis or EMT. TXL attenuated renal fibrosis and decreased the expressions of TGF-*β*1 and *α*-SMA in unilateral ureteral occlusion mice [16]. Ginsan, a polysaccharide extracted from* Panax ginseng*, significantly suppressed the accumulations of TGF-*β*, collagen, and *α*-SMA [17]. Paeoniflorin (PF), the key active constituent of* Paeonia lactiflora *Pallas, has previously been reported to prevent the progression of renal fibrosis in UUO mice. The antifibrosis efficacy of PF was mainly reflected in improving histopathological disorders and reducing collagen deposition in kidney tissues [18]. Additionally, PF successfully downregulated TGF-*β*1 expression and inhibited smad2/3 activation in fibrotic kidneys induced by UUO [18]. These findings strongly support the hypothesis that TXL may attenuate EMT in DN.

The aim of the present study was to explore the mechanisms of TXL-mediated renoprotection and determine whether TXL can inhibit TGF-*β*1-induced EMT in DN. Results showed that TXL successfully inhibited TGF-*β*1 expression and TGF-*β*1-induced EMT, and TXL may be a new possible therapy in diabetic nephropathy.

## 2. Material and Methods

### 2.1. Preparation of Tong xinluo Ultrafine Powder Solution

Tong xinluo ultrafine powder was procured by Shi-jiazhuang Yiling Pharmaceutical company (Lot no. 071201, Shi-jiazhuang Yiling Pharmaceutical Co., Shijiazhuang, China). It contains a group of medicines such as* Panax ginseng *C. A. Mey. Extract and* Paeonia lactiflora *Pall. extract. The detailed formulation of TXL is shown in Supplementary Materials available online at http://dx.doi.org/10.1155/2014/123497. The herbal drugs were authenticated and standardized on marker compounds according to the Chinese Pharmacopoeia (2005). To reduce the dose variability of TXL among different batches, the species, origin, harvest time, medicinal parts, and concocted methods for each component were strictly standardized. Moreover, high performance liquid chromatography (HPLC), high performance capillary electrophoresis, and gas chromatography were applied to quantitate the components of TXL. HPLC was taken to check fingerprint chromatograms of the aqueous extracts of the 10 batches for similarity analysis [12]. The detailed result of HPLC can be found in Supplementary Material. The herbal drugs were ground to ultrafine powder with the diameter ≤10 *μ*m by a micronizer. In vivo experiment, 750 mg/kg TXL ultrafine powder dissolved in aquadistillate was intragastrically administered each day. In cell culture, TXL ultrafine powder was dissolved in serum-free DMEM/F12 (Dulbecco's modified Eagle's medium/F12). The solution was sonicated for 1 hour followed by centrifugation at 1164 g for 10 min. The supernatant was centrifuged for a second time. Finally, it was filtrated by 0.22 *μ*m filters and stored at −20°C. Meanwhile, the precipitate was heated and dried at 60°C in order to calculate the practical volume of dissolved Tong xinluo ultrafine powder. The final concentration is 2000 *μ*g/mL [19]. In in vitro experiment, it was diluted with DMEM/F12.

### 2.2. Animals

To explore the effect of TXL on TGF-*β*1 expression and renal tubular EMT in DN, KK-Ay mice were spontaneous animal models for the evaluation of pathogenesis and treatment in patients with DN [20]. DN was diagnosed when their random blood glucose (RBG) was ≥16.7 mmol/L and urine albumin creatine ratio (ACR) was ≥300 *μ*g/mg. C57BL/6J mice were fed with common forage and KK-Ay mice were fed with high fat forage for 4 weeks. C57BL/6J mice gavaged with aquadistillate were set as normal control group (Normal, *n* = 10), KK-Ay mice gavaged with aquadistillate were set as diabetic nephropathy group (DN group, *n* = 10), and KK-Ay mice treated with TXL (750 mg/kg/per day, gavage) were set as TXL group (DN + TXL group, *n* = 10). After being treated for 12 weeks, blood and 24-hour urine were collected. Renal tissue was immediately frozen for western blot and RT-PCR and 4% (w/v) paraformaldehyde was used to fix renal tissues for immunofluorescence staining. C57BL/6J and KK-Ay mice were purchased from the Chinese Academy of Medical Sciences (8 weeks of age, Beijing, China). The experiment complied with the Animal Management Rule of the Ministry of Public Health, China, and the experimental protocol was approved by the Animal Care Committee of Capital Medical University, Beijing, China.

### 2.3. Cell Culture

Human renal tubuloepithelial cells (HKCs) were purchased from Cell Resource Center in China. It was incubated with Dulbecco's modified Eagle's medium/F12 (DMEM/F12) containing 10% (v/v) fetal calf cerum (FBS). Cells were maintained at 37°C in a 5% (v/v) CO_2_ water-saturated atmosphere. Recombinant human transforming growth factor *β*1 (TGF-*β*1, eBioscience, Santiago, USA) was used to induce EMT in HKCs in vitro. Five concentrations (5 ng/mL–25 ng/mL) of TGF-*β*1 and five time points (24–72 hours) were applied to observe the effect of TGF-*β*1 on EMT in vitro. Concentration of 10 ng/mL of TGF-*β*1 was used to induce EMT in the following study. Cells exposed to 10 ng/mL TGF-*β*1 for 48 hours were set as TGF-*β*1 group. In order to test the TXL-mediated protection in EMT, we first preconditioned the HKCs with TXL for 30 min before they were exposed to TGF-*β*1 [8]. Cells exposed to 10 ng/mL TGF-*β*1 and 250 *μ*g/mL TXL for 48 hours were set as TGF-*β*1 + TXL group. Cells cultured in normal DMEM/F12 without TGF-*β*1 were set as normal group.

### 2.4. RT-PCR Analysis

Total RNA was extracted with Trizol (TIANGEN, Beijing, China) in accordance with the manufacturer's recommendations. Each sample was reverse transcribed into cDNA using SuperScript II Reverse Transcriptase (Invitrogen). Gene expression was quantified by means of the comparative Ct method (ΔΔCt) and the relative quantification (RQ) was calculated as 2^−ΔΔCt^ [21–23]. Relative mRNA levels of E-CA, *α*-SMA, smad3, smad7, Col IV, and FN were examined and normalized to *β*-actin mRNA. All RT-PCRs were performed in triplicate, and the data was presented as mean ± SD. The primers of TGF-*β*1, E-CA, *α*-SMA, smad3, smad7, Col IV, and FN are in Table 1.

### 2.5. Western Blot Analysis

TGF-*β*1, E-CA, *α*-SMA, smad3/p-smad3, and smad7 were detected by western blot as previously described [24, 25]. Western blot was performed with mouse monoclonal to TGF-*β*1 (Abcam, 1 : 500), rabbit monoclonal to E-CA (CST, 1 : 500), rabbit polyclonal to *α*-SMA (Abcam, 1 : 500), rabbit monoclonal to smad3 (Abcam, 1 : 500), rabbit polyclonal to p-smad3 (CST, 1 : 500), and rabbit polyclonal to smad7 (Epitomic, 1 : 500). The membranes were then incubated with horseradish peroxidase-conjugated secondary antibody (1 : 5000). Western blot analyses were performed at least in triplicate. Densitometry was detected by Image J [26, 27].

### 2.6. Immunofluorescence Staining

Renal tissue sections were prepared after fixation in 4% (w/v) paraformaldehyde and embedded in paraffin. For semiquantitative analysis, 20 high-power microscope fields of renal tissue were randomly selected. The mean fluorescence activity was analyzed by image-pro plus 6.0 software [28, 29]. Cells at 80% confluence on coverslips were fixed with 4% (w/v) paraformaldehyde. For semiquantitative analysis, 40 high-power microscope fields of cells were randomly selected, and the mean fluorescence activity was analyzed with image-pro plus 6.0 software [28, 29]. Antibodies and dilutions were as follows: rabbit monoclonal to E-CA (CST, 1 : 50), rabbit polyclonal to *α*-SMA (Abcam, 1 : 50), rabbit polyclonal to FN (Abcam, 1 : 250), and rabbit polyclonal to Col IV (Abcam, 1 : 250). DAPI was used to stain the cell nuclei (blue). Confocal microscope (Leica TCS SP5 MP, Heidelberg GmbH, Germany) was used in this experiment.

### 2.7. Biochemical Assays and Light Microscopy

Blood was drawn from mice fasting overnight at 24 weeks of age, and mouse metabolic cages were used to collect urine samples of 24 hours. Tissue for light microscopy was fixed in 4% (w/v) paraformaldehyde and then embedded in paraffin. Four-micrometer thick sections of renal tissue were processed for hematoxylin-eosin (HE) and Masson's trichrome staining.

### 2.8. Statistical Analysis

The results were presented as means ± SD of at least three independent experiments. Statistical analysis was carried out using SPSS17.0. Data were analyzed with the one-way ANOVA. *P* < 0.05 was considered statistically significant.

## 3. Results

### 3.1. Effects of TXL on TGF-*β*1 Expression and EMT Markers in Renal with DN

**Figure 1:**
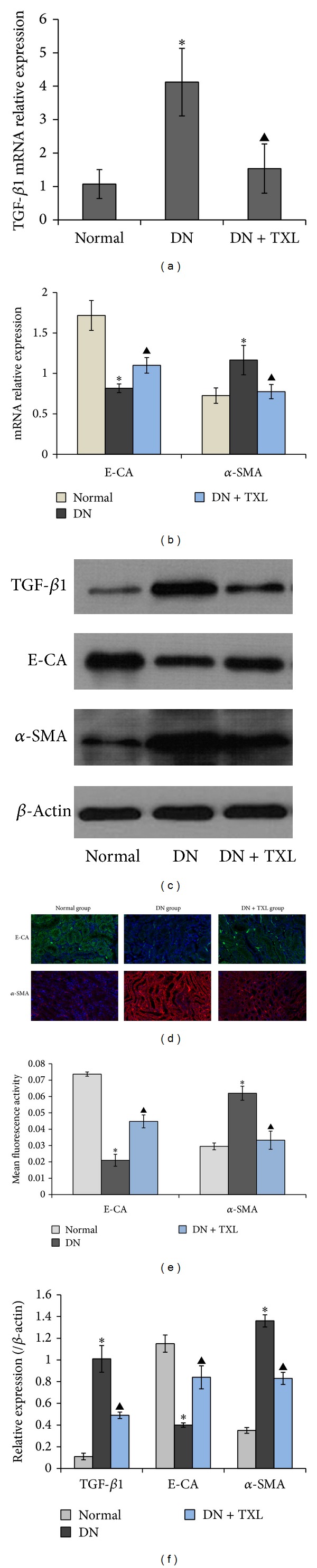
Effects of TXL on the expressions of TGF-*β*1, E-CA, and *α*-SMA in renal tissues of KK-Ay mice. (a) mRNA expression of TGF-*β*1 was determined by RT-PCR with *β*-actin as an internal control. (b) mRNA expressions of E-CA and *α*-SMA were determined by RT-PCR with *β*-actin as an internal control. (c) Representative bands of TGF-*β*1, E-CA, and *α*-SMA detected by western blot. (d) Representative immunofluorescence staining photographs of E-CA and *α*-SMA, visualized by confocal microscope. Images are shown at 20x. (e) Mean fluorescence activity of E-CA and *α*-SMA analyzed by image-pro plus 6.0 software. (f) Densitometry analysis of TGF-*β*1, E-CA, and *α*-SMA bands from (c), normalized to *β*-actin.

Overwhelming evidences implicate that TGF-*β*1 acts as the key mediator of tubular EMT [30]. To observe whether TXL affected TGF-*β*1 expression and markers of EMT in vivo, RT-PCR was first performed to examine the expression of TGF-*β*1 at 24 weeks of age. RT-PCR results showed that TGF-*β*1 was significantly enhanced in DN group compared with normal group (Figure 1(a), *P < 0.05). After being treated with TXL for 12 weeks, expression of TGF-*β*1 was significantly decreased compared with DN group (Figure 1(a), ^▲^ *P* < 0.05). Additionally, Western blot results were consistent with RT-PCR results (Figures 1(c) and 1(f)). TXL suppressed TGF-*β*1 expression both at mRNA and protein levels. Next, to further verify the effect of TXL on tubular EMT, E-CA and *α*-SMA were detected by RT-PCR, western blot, and immunofluorescence staining, respectively. RT-PCR and western blot results showed that *α*-SMA was enhanced and E-CA was decreased in DN group compared with normal group (Figures 1(b), 1(c), and 1(f), *P < 0.05). More importantly, TXL significantly decreased *α*-SMA expression and increased E-CA expression compared with DN group both at mRNA and protein levels (Figures 1(b), 1(c), and 1(f), ^▲^ *P* < 0.05). Immunofluorescence staining showed the similar results that epithelial marker E-CA was significantly decreased, while *α*-SMA was increased in DN group compared with normal group (Figures 1(d) and 1(e), *P < 0.05). More importantly, TXL treatment significantly restored E-CA and *α*-SMA expressions (Figures 1(d) and 1(e), ^▲^ *P* < 0.05). These results demonstrated that TXL can inhibit TGF-*β*1 expression and EMT in DN.

### 3.2. TGF-*β*1 Induces Epithelial-to-Mesenchymal Transition In Vitro

**Figure 2:**
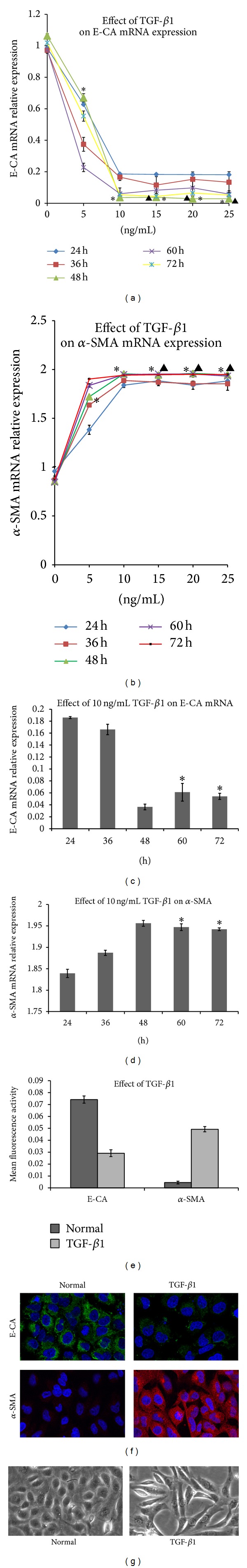
Time-course and dose-response of E-CA and *α*-SMA mRNA expressions in HKCs induced by TGF-*β*1. (a) HKCs were treated with five concentrations (5 ng/mL–25 ng/mL) of TGF-*β*1, and expression of E-CA mRNA was determined by RT-PCR with *β*-actin as an internal control. (b) Expression of *α*-SMA mRNA was determined by RT-PCR with *β*-actin as an internal control. (c) E-CA mRNA expression of cells exposed to 10 ng/mL TGF-*β*1 from 24 to 72 hours, normalized to *β*-actin. (d) *α*-SMA mRNA expression of cells exposed to 10 ng/mL TGF-*β*1 from 24 to 72 hours, normalized to *β*-actin. (e) Mean fluorescence activity of E-CA and *α*-SMA in normal HKCs and cells incubated with 10 ng/mL TGF-*β*1 for 48 hours. Photographs were analyzed by image-pro plus 6.0 software.(f) Representative immunofluorescence staining photographs of E-CA and *α*-SMA in normal HKCs and cells incubated with 10 ng/mL TGF-*β*1 for 48 hours. Images are shown at 40x and visualized by confocal microscope. (g) Representative photographs of phenotype change in HKCs induced by 10 ng/mL TGF-*β*1 compared with normal cells.

Five concentrations (5 ng/mL–25 ng/mL) of TGF-*β*1 and five time points (24–72 hours) were applied to observe the effect of TGF-*β*1 on EMT in vitro. Increase of *α*-SMA and loss of E-CA are the markers of EMT. RT-PCR and immunofluorescence staining were used to show the changes of EMT markers. RT-PCR showed a dose-dependent manner in the decrease of E-CA mRNA, lowest at 10 ng/mL (Figure 2(a), *P < 0.05 versus normal, ^▲^ *P* > 0.05 versus 10 ng/mL), while *α*-SMA mRNA was significantly increased and peaked at 10 ng/mL (Figure 2(b), *P < 0.05 versus normal, ^▲^ *P* > 0.05 versus 10 ng/mL). Cells exposed to 10 ng/mL TGF-*β*1 exhibited a time-dependent manner in the decrease of E-CA mRNA, lowest at 48 hours, while *α*-SMA mRNA was significantly increased and peaked at 48 hours (Figures 2(c) and 2(d), *P > 0.05 versus 48 hours). Additionally, the results of immunofluorescence staining showed that fluorescence activity of *α*-SMA was obviously enhanced and E-cadherin was decreased in TGF-*β*1 group compared with normal group (Figures 2(e) and 2(f), *P* < 0.05). TGF-*β*1 also induced an elongated and fibroblast-like phenotype change in HKCs (Figure 2(g)). These changes suggested that the HKCs had begun to lose the epithelial phenotype and changed to express the myofibroblastic markers.

### 3.3. Effects of TXL on TGF-*β*1-Induced Epithelial-to-Mesenchymal Transition In Vitro

**Figure 3:**
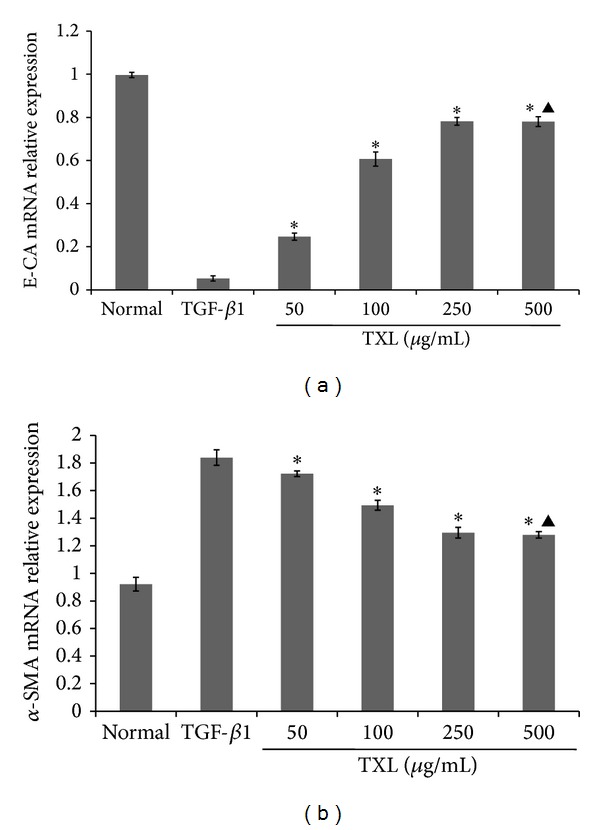
Effects of different concentrations of TXL on the expressions of E-CA and *α*-SMA in HKCs. (a) Cells were treated with different concentrations of TXL (50–500 *μ*g/mL) for 48 hours, and E-CA mRNA expression was determined by RT-PCR with *β*-actin as an internal control. (b) Cells were treated with different concentrations of TXL (50–500 *μ*g/mL) for 48 hours, and *α*-SMA mRNA expression was determined by RT-PCR with *β*-actin as an internal control.

To determine whether TXL affects EMT in vitro, markers of EMT (*α*-SMA and E-CA) were examined by RT-PCR. HKCs were treated with different concentrations of TXL (50–500 *μ*g/mL). 10 ng/mL TGF-*β*1 was selected to induce EMT. RT-PCR showed that TXL restored E-CA expression in a dose-dependent manner, peaked at 250 *μ*g/mL (Figure 3(a), *P < 0.05 versus TGF-*β*1 group, ^▲^ *P* > 0.05 versus 250 *μ*g/mL), whereas *α*-SMA expression was inhibited and lowest at 250 *μ*g/mL (Figure 3(b), *P < 0.05 versus TGF-*β*1 group, ^▲^ *P* > 0.05 versus 250 *μ*g/mL). TXL positively reversed TGF-*β*1-induced downregulation of E-CA and upregulation of *α*-SMA.

### 3.4. Effects of Tong xinluo on TGF-*β*1/Smads Signal Pathway In Vivo and Vitro

**Figure 4:**
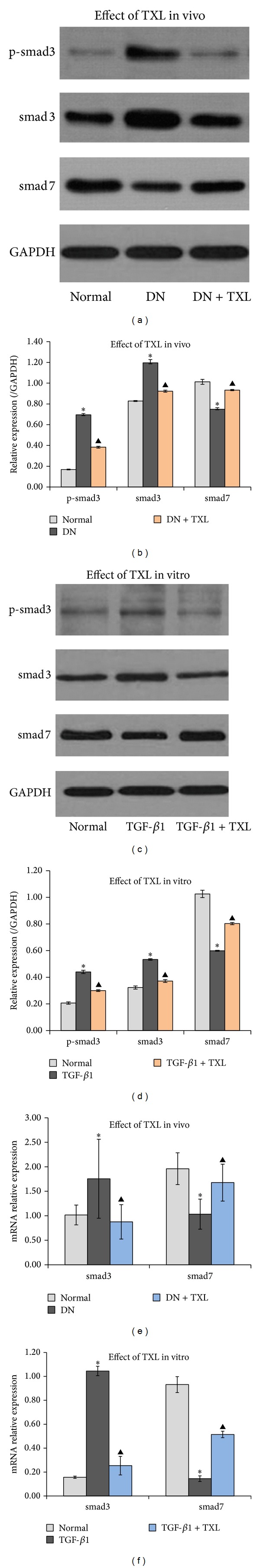
Effects of TXL on the expressions of p-smad3/smad3 and smad7 in both renal tissues and HKCs. (a) Representative bands of p-smad3/smad3 and smad7 detected by western blot of renal tissues. (b) Densitometry analysis of p-smad3/smad3 and smad7 bands from (a), normalized to GAPDH. (c) Representative bands of p-smad3/smad3 and smad7 detected by western blot of HKCs. (d) Densitometry analysis of p-smad3/smad3 and smad7 bands from (b), normalized to GAPDH. (e) Effects of TXL on smad3 and smad7 mRNA expressions in renal tissues, normalized to *β*-actin. (f) Effects of TXL on smad3 and smad7 mRNA expressions in HKCs, normalized to *β*-actin.

In EMT, various studies have explored the roles ofTGF-*β*1 in activating smads [31]. To explore whether TXL affects TGF-*β*1/smads signal pathway, renal tissue and HKCs were detected by RT-PCR and western blot, respectively. 10 ng/mL TGF-*β*1 was applied to induce EMT in HKCs. Cells exposed to 10 ng/mL TGF-*β*1 were set as TGF-*β*1 group, and cells exposed to 10 ng/mL TGF-*β*1 and 250 *μ*g/mL TXL were set as TGF-*β*1 + TXL group. The normal cells cultured only with DMEM/F12 were set as normal group. In vivo, western blot showed that p-smad3/smad3 were remarkably increased and smad7 was decreased in DN group compared with normal group (Figures 4(a) and 4(b), *P < 0.05), while p-smad3 and smad3 were significantly decreased and smad7 was increased in TXL group compared with DN group (Figures 4(a) and 4(b), ^▲^ *P* < 0.05). RT-PCR results were consistent with western blot results (Figure 4(e)). Additionally, in vitro, western blot and RT-PCR also showed the significant increase of p-smad3 and smad3 in TGF-*β*1 group compared with normal group. In contrast, smad7 was significantly decreased (Figures 4(c), 4(d), and 4(f), *P < 0.05). Importantly, p-smad3/smad3 were remarkably decreased and smad7 was increased in the TGF-*β*1 + TXL group compared with TGF-*β*1 group (Figures 4(c), 4(d), and 4(f), ^▲^ *P* < 0.05). Thus, the present results showed that TXL positively deceased p-smad3/smad3 expressions and increased the expression of smad7.

### 3.5. Prevention of Enhanced Collagen IV and Fibronectin Expressions by Tong xinluo

**Figure 5:**
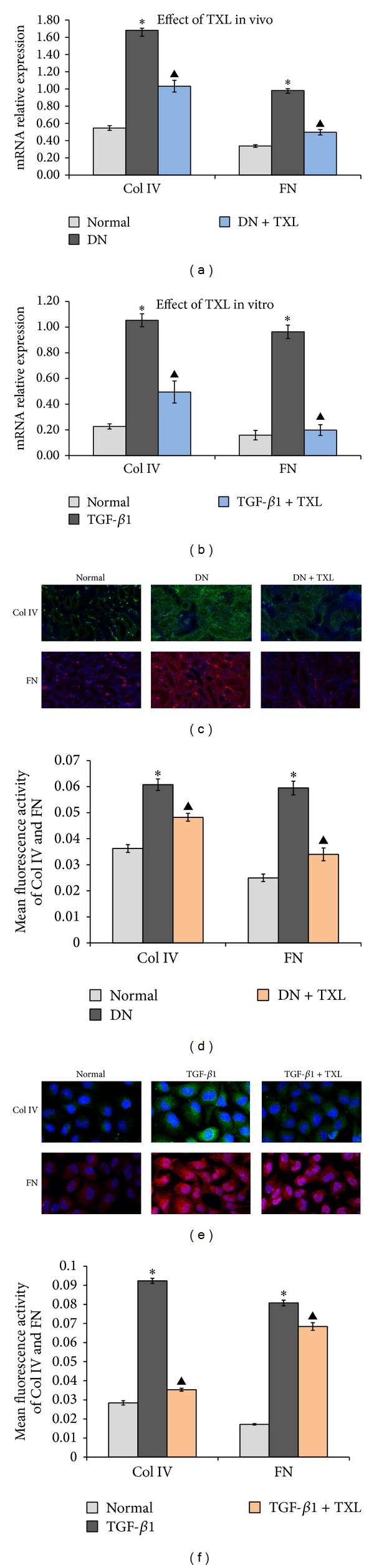
Effects of TXL on the expressions of Col IV and FN. (a) Col IV and FN mRNA expressions were detected by RT-PCR in vivo, normalized to *β*-actin. (b) Col IV and FN mRNA expressions were detected by RT-PCR in vitro, normalized to *β*-actin. (c) Representative photographs of Col IV and FN by immunofluorescence staining in renal tissues. Images are shown at 20x. (d) Mean fluorescence activity of Col IV and FN in renal tissues. Photographs were analyzed by image-pro plus 6.0 software. (e) Representative photographs of cells stained with primary antibody against Col IV and FN together with DAPI (blue). Images are shown at 40x. (f) Mean fluorescence activity of Col IV and FN detected by immunofluorescence staining. Photographs were analyzed by image-pro plus 6.0 software.

EMT paves the way for extracellular matrix (ECM) deposition and ultimately renal fibrosis [32]. To explore the effect of TXL on ECM, RT-PCR was used to detect the changes of collagen IV (Col IV) and fibronectin (FN) mRNA. In vivo, results showed that Col IV and FN mRNA were markedly increased in DN group compared with normal group (Figure 5(a), *P < 0.05), while Col IV and FN mRNA were significantly decreased in TXL group compared with DN group (Figure 5(a), ^▲^ *P* < 0.05). Meantime, results of immunofluorescence staining also exhibited that Col IV and FN proteins were observably increased in DN group compared with normal group (Figures 5(c) and 5(d), *P < 0.05). Importantly, TXL successfully decreased the expressions of Col IV and FN protein (Figures 5(c) and 5(d), ^▲^ *P* < 0.05). Furthermore, in vitro, results showed that TXL significantly decreased the expressions of Col IV and FN mRNA compared with TGF-*β*1 group (Figure 5(b), ^▲^ *P* < 0.05). Immunofluorescence staining of HKCs showed the same results as RT-PCR (Figures 5(e) and 5(f), ^▲^ *P* < 0.05). Importantly, the results of Col IV and FN expressions in vitro were consistent with the results in vivo. The present results suggested that TXL may have the function of ameliorating ECM deposition.

### 3.6. Effects of TXL on 24 h-UAER, BUN, and Creatine Clearance Ratio and Morphological Changes

**Figure 6:**
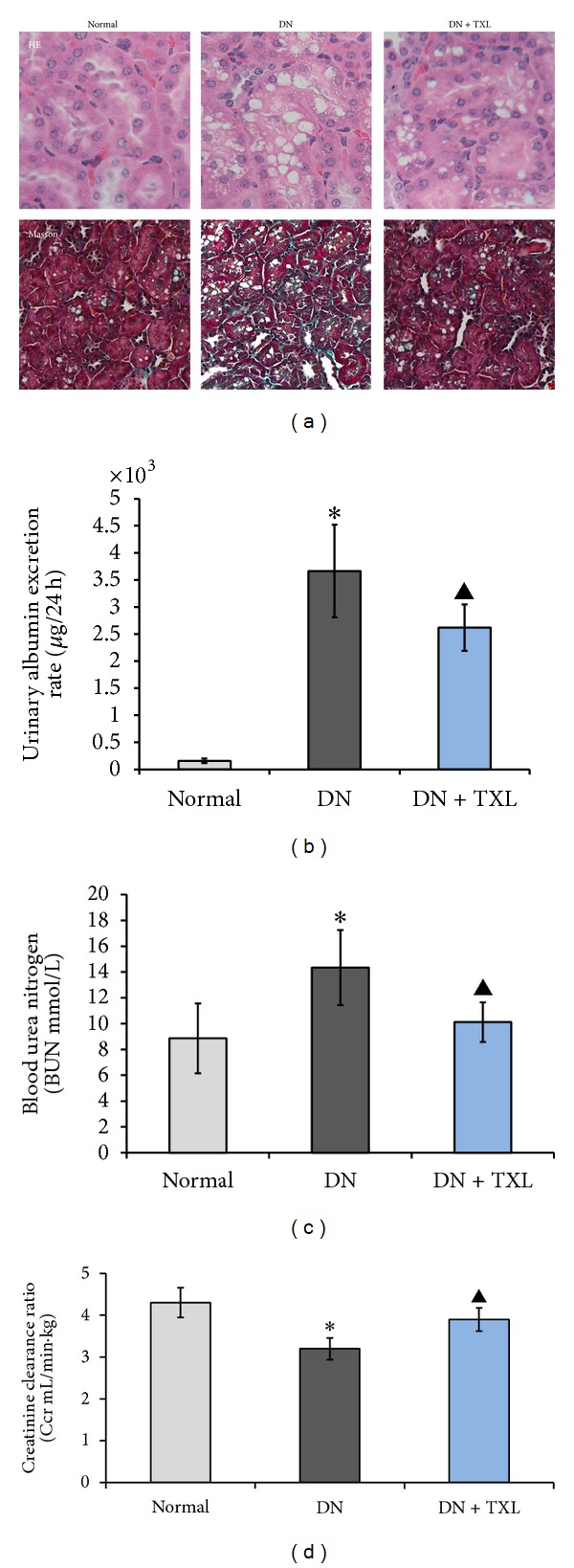
Effects of TXL on renal function and structure. (a) HE staining and Masson staining of renal tissues. HE staining showed the vacuoles degeneration of renal tubular epithelial cells in DN group and TXL partly ameliorated it. Masson staining exhibited the deposition of collagen fibers in DN group and TXL effectively alleviated it. (b) Effect of TXL on urine albumin excretion. (c) Effect of TXL on BUN. (d) Effect of TXL on creatinine clearance ratio.

Elevated urine albumin excretion ratio (UAER) is a key risk factor for renal and cardiovascular disease in type 2 diabetes [33]. Creatinine clearance ratio (Ccr) is generally taken as an evaluation of renal filtration function. Renal morphology was observed by light microscopy at 24 weeks of age. Compared with the normal group, 24 h-UAER and blood urea nitrogen (BUN) in DN group were significantly increased, while Ccr was decreased (Figures 6(b), 6(c), and 6(d), *P < 0.05). Interestingly, 24 h-UAER and BUN were decreased and Ccr was increased in TXL group compared with the DN group (Figures 6(b), 6(c), and 6(d), ^▲^ *P* < 0.05). HE staining showed the vacuoles degeneration of renal tubular epithelial cells in DN group and TXL partly ameliorated it. Masson staining exhibited the amount of collagen fibers and TXL positively mitigated these pathological variations compared with DN group (Figure 6(a)). The present results suggested that TXL exerted its positive effect in DN via decreasing UAER/BUN and increasing Ccr. Furthermore, it ameliorated renal structure.

## 4. Discussion

Diabetic nephropathy (DN), a leading cause of mortality in diabetic patients, affects approximately one-third of all diabetic patients and causes a heavy economic burden for them [34]. The crucial pathology underlying DN is interstitial fibrosis [25]. EMT of tubuloepithelial cells is a widely recognized mechanism that sustains interstitial fibrosis in DN [35]. EMT can be induced or regulated by various growth and differentiation factors, including TGF-*β*1 and connective tissue growth factor (CTGF). Among these, TGF-*β*1 has received much attention as a major inducer of EMT during fibrosis [31]. In the present study, RT-PCR results exhibited that TGF-*β*1 expression was significantly enhanced in DN group and pointed to the crucial role of TGF-*β*1 in DN. The present result was consistent with previous reports [36, 37]. Additionally, we chose to examine several well-established typical events of EMT in renal tissue and renal tubuloepithelial cells that have been widely used in the study of TGF-*β*1-induced EMT [25, 38]. Accompanied with the increase of TGF-*β*1 expression, the expression of E-CA was significantly decreased while *α*-SMA was elevated in DN group. In cell experiment, TGF-*β*1 evoked EMT in HKCs was obtained by assessment of morphological changes, increased expression of *α*-SMA, and down-regulation of the epithelial marker E-cadherin. More importantly, we present novel findings that TGF-*β*1 is able to induce EMT in a dose and time dependent manner. Cells exposed to 10 ng/mL TGF-*β*1 for 48 hours were used in vitro experiment to induce EMT.

Chinese medicine Tong xinluo (TXL) is extracted from a group of herbal medicines including* Panax ginseng *C. A. Mey. extract,* Paeonia lactiflora *Pall. extract, and* Borneolum syntheticum *[39]. Some active components extracted from* Panax ginseng* significantly suppressed TGF-*β* expression [17].* Paeoniflorin* can exert antifibrogenic effects by downregulating smad3 expression and phosphorylation through TGF-*β*1 signaling [40]. According to the potentially advantageous regulation of cell signaling events by components of TXL in kidney [16, 18], we tried to explore whether TXL could inhibit EMT in DN. Importantly, we now demonstrate that TXL strikingly inhibits TGF-*β*1 expression in DN and also inhibits TGF-*β*1-induced morphological and phenotypic changes in HKCs. RT-PCR results showed that TXL greatly decreased *α*-SMA mRNA expression and increased E-CA mRNA expression. The inhibition of E-CA downregulation and *α*-SMA upregulation seen in the current studies was maximal with 250 *μ*g/mL concentration of TXL. This pharmacological mechanism of TXL inhibiting EMT is absolutely in accordance with previous studies; Hung et al. demonstrated that counteract TGF-*β*1 and its downstream signal transducers, like smad3 and smad7, may be an effective therapy in inhibiting EMT [24, 41]. The present study also showed that TXL is able to negatively modulate TGF-*β*1 signaling at several levels. Having demonstrated that TXL blocked TGF-*β*1 expression and TGF-*β*1-evoked changes in the cell phenotype, we examined the ability of TXL to interfere with these TGF-*β*1 signaling elements. TGF-*β*1 caused upregulation of smad3 and p-smad3 expressions, while decreased smad7 expression. More importantly, the changes of smads were restored to a greater or lesser extent by TXL, reflected in the decrease of smad3/p-smad3 expressions and the increase of smad7 expression in TXL group. Previous works had demonstrated the involvement of smads in TGF-*β*1-mediated EMT and proven the positive effect of regulating these smads in inhibiting EMT. TXL appears to inhibit EMT via decreasing TGF-*β*1-induced smad3 and p-smad3 expression, which is consistent with the previous studies [24, 42]. smad7 was a negative regulator of TGF-*β*1-induced EMT [43]. In the present study, western blot and RT-PCR results showed that TXL significantly increased smad7 expression both in vitro and in vivo. The most possible reason of TXL inhibiting EMT may be that TXL represses TGF-*β*1 expression and blocks downstream signaling cascades of TGF-*β*1 in HKCs. Taken together, the present results demonstrate that TXL may be a positive therapy in inhibiting TGF-*β*1-induced EMT. These results further support TXL to be of therapeutic potential in treating diabetic nephropathy via inhibiting EMT.

Excessive deposition of extracellular matrix (ECM) is a histological hallmark of DN, which is closely related to the progressive decline of renal function [44].* Panax ginseng* is the primary component of TXL; researches showed that diabetes-induced upregulations of ECM proteins in the kidneys were significantly diminished by* Panax ginseng* administration; furthermore, albuminuria in the diabetic mice was prevented [45]. However, whether TXL ameliorated renal structure and function by inhibiting ECM accumulation in TGF-*β*1-induced EMT remains unclear. The present findings indeed supported the notion that TXL suppressed expressions of Col IV and FN both at mRNA and protein levels. In this sense, present results may account for part of the mechanisms that TXL improve renal structure and function. Indeed, we observed a significant decrease of 24 h-UAER and BUN, while Ccr was increased in TXL group, as compared to untreated diabetic mice. These findings suggest that administration of TXL may contribute to tubular repair and may accelerate the decay in renal function observed in the diabetic condition. The current results demonstrate that TXL successfully ameliorates renal structure and function, and its novel therapeutic potential in diabetic nephropathy is highly attractive.

## 5. Conclusion

In summary, the present data provides a new perspective on the molecular effects of TXL on DN by showing that TXL treatment inhibits TGF-*β*1-induced EMT. But more basic researches and clinical studies are needed to future investigate the positive role of TXL in protecting DN. Moreover, the present data inspires further study to explore the effects and mechanisms of Chinese herbal compounds, which may have important therapeutic applicability in DN.

## Supplementary Material

Tongxinluo, a Chinese herbal compound, includes a group of medicines such as *Panax ginseng* C. A. Mey. extract, *Paeonia lactiflora* Pall. extract. It is a mixture of plant and animal products, and the detailed formulation is as follows.

## Figures and Tables

**Table 1:** Oligonucleotide primers used in the study.

Gene name	Forward	Reverse
mmu-TGF-*β*1	5′-atacgcctgagtggctgtct-3′	5′-ctgatcccgttgatttcca-3′
hsa-E-CA	5′-tcttcggaggagagcggtggtcaaa-3′	5′-gccgagcgtccaggcccctgtgcag-3′
mmu-E-CA	5′-gagtggagaacgaggaaccctttga-3′	5′-acgtgtccggctctcgagcggtata-3′
hsa-*α*-SMA	5′-atcaaggagaaactgtgttatgtag-3′	5′-gatgaaggatggctggaacagggtc-3′
mmu-*α*-SMA	5′-gagtcagcgggcatccacgaaa-3′	5′-tgctgggtgcgagggctgtgat-3′
hsa-smad3	5′-gaggcgtgcggctctactacatc-3′	5′-gccaggagggcagcgaact-3′
mmu-smad3	5′-gcacagccaccatgaattac-3′	5′-gcacagccaccatgaattac-3′
hsa-smad7	5′-aggtgttccccggtttctcca-3′	5′-ttcacaaagctgatctgcacggt-3′
mmu-smad7	5′-gctttcagattcccaacttctt-3′	5′-gatatccagggagggctcttg-3′
hsa-Col IV	5′-tggtcttactgggaactttgctgc-3′	5′-ggtgggatctgaatggtctggc-3′
mmu-Col IV	5′-tggtcttactgggaactttgctgc-3′	5′-accctgtggtccaacgactcctctc-3′
hsa-FN	5′-agaagtgggaccgtcagggaga-3′	5′-caggagcaaatggcaccgaga-3′
mmu-FN	5′-tctgggaaatggaaaaggggaatgg-3′	5′-cactgaagcaggtttcctcggttgt-3′
