# Supplementary material for: Effects of Chinese Medicine Tong xinluo on Diabetic Nephropathy via Inhibiting TGF-β1-Induced Epithelial-to-Mesenchymal Transition
Source: Evid Based Complement Alternat Med. 2014 Apr 17;2014:123497. doi: 10.1155/2014/123497 (PMC4016864; doi:10.1155/2014/123497)
Supplement: Supplementary file 1 — Tongxinluo, a Chinese herbal compound, includes a group of medicines such as Panax ginseng C. A. Mey. extract, Paeonia lactiflora Pall. extract. It is a mixture of plant and animal products, and the detailed formulation is as follows. [file 123497.f1.pdf]

Formulation of Tong xinluo

Ingredient	Components	%
Panax ginseng C. A. Mey. extract	Root and rhizome	1.7
Paeonia lactiflora Pall. extract	Root	1.6
Ziziphus jujuba Mill. Var. *spimosa* (Bunge) extract	Seed	1.2
Santalum album. extract	Heartwood of stem	0.4
Dalbergia odorifera extract	Heartwood of stem and root	4.0
Steleophaga plancyi (Boleny) Wingless cockroach (beetle)	Female dried body	18.1
Scolopendra subspinipes mutilans L. Koch red-headed centipede	Dried body	3.6
Hirudo nipponica Whitman leech	Dried body	27.3
Cryptotympana pustulata Fabricius cicada	Skin	18.1
Buthus martensii Karsch scorpion	Dried body	18.1
Boswellia carteri	Resin	6.0
Borneolum syntheticum	$C10H18O$	3.6

Result of high performance liquid chromatography (HPLC)

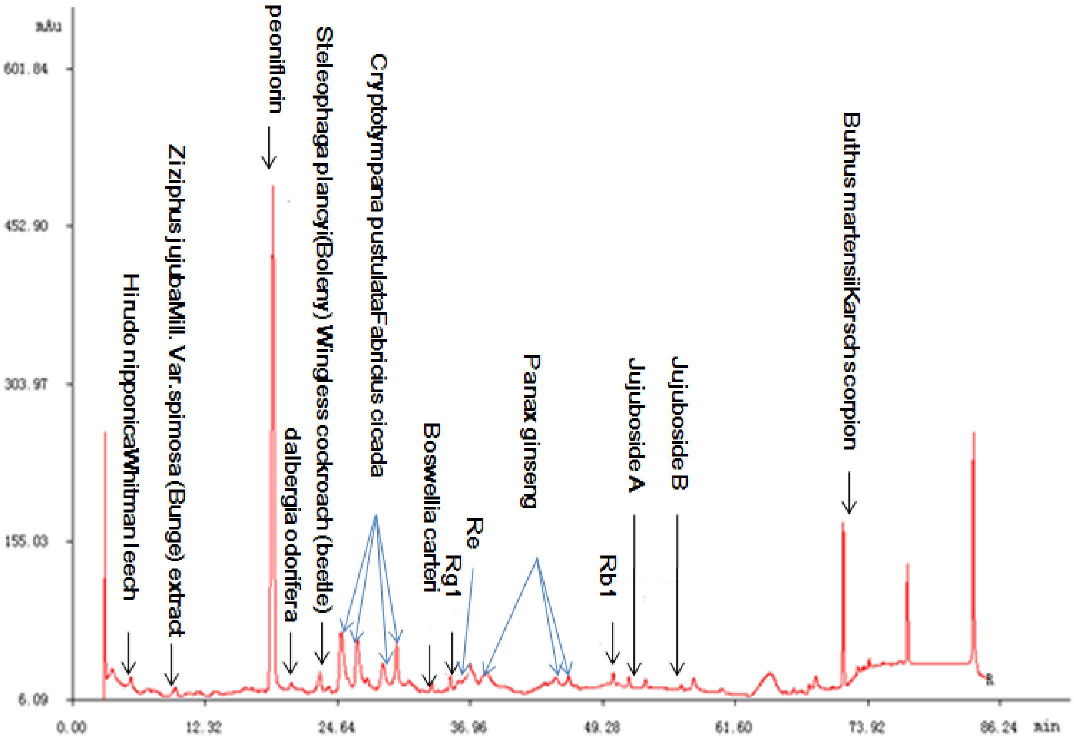

High performance liquid chromatography (HPLC) of Tongxinluo
